# Multiscale 3D microfluidic platform for intraorganoid delivery

**DOI:** 10.21203/rs.3.rs-8436544/v1

**Published:** 2026-01-27

**Authors:** Maria Jose Quezada, Jamin Lee, Zengyao Lv, Khizar Nandoliya, Ingrid Cheung, Qiong Wang, Woo-Yeol Maeng, Naijia Liu, Amir Vahabikashi, Kyoung-Ho Ha, Yong-Woo Kang, Dae-Hyeon Song, Yuming Huang, Clara Asseily, Rakan Walid da Cruz, Shreyaa Khanna, Jintao Liu, John D. Finan, Lara Leoni, Daniele Procissi, Yonggang Huang, Gyu-Chul Yi, Juyeol Bae, John A. Rogers, Colin K. Franz

**Affiliations:** 1 Regenerative Neurorehabilitation Laboratory, Shirley Ryan Ability Lab, Chicago, IL 60611, USA.; 2 Department of Biomedical Engineering, Northwestern University, Evanston, IL 60208, USA.; 3 Department of Physical Therapy and Human Movement Sciences, Northwestern University, Chicago, IL 60611, USA.; 4 Interdisciplinary Program in Neuroscience, College of Science, Seoul National University, Seoul, 08826, South Korea.; 5 Center for Novel Epitaxial Quantum Architectures, Department of Physics and Astronomy, Seoul National University, Seoul, 08826, South Korea.; 6 Querrey Simpson Institute for Bioelectronics, Northwestern University, Evanston, IL, 60208, USA.; 7 Department of Civil and Environmental Engineering, Northwestern University, Evanston, IL 60208, USA.; 8 Department of Neurological Surgery, Feinberg School of Medicine, Northwestern University, Chicago, IL 60611, USA.; 9 Department of Physical Medicine and Rehabilitation, Northwestern University.; 10 Bioengineering Department and Institute for Mechanobiology, Northeastern University, Boston, MA, 02115, USA.; 11 Department of Materials Science and Engineering, Korea Advanced Institute of Science and Technology, Daejeon, 34141, South Korea.; 12 Department of Mechanical Engineering, Chonnam National University, 77 Yongbong-ro, Buk-gu, Gwangju 61186, Republic of Korea.; 13 Advanced Medical Device Research Center for Cardiovascular Disease, Chonnam National University, 77 Yongbong-ro, Buk-gu, Gwangju 61186, Republic of Korea; 14 Department of Mechanical and Industrial Engineering, University of Illinois Chicago, Chicago, IL 60607, USA.; 15 Department of Radiology, Feinberg School of Medicine, Northwestern University, Chicago, IL 60611, USA.; 16 Department of Materials Science and Engineering and Department of Mechanical Engineering, Northwestern University, Evanston, IL 60208, USA.; 17 The Ken and Ruth Davee Department of Neurology, Feinberg School of Medicine, Northwestern University, Chicago, IL 60611, USA.

## Abstract

Neural organoids are emerging as advanced three-dimensional (3D) *in vitro* models for recapitulating human development and pathology, but they lack dedicated mass-transport pathways that perfuse interior regions, limiting control over solute concentrations in deep tissues. Microfluidic technologies hold promise for intra-organoid delivery, yet creating high-resolution 3D transport architectures spanning arteriole-to-venule scales while integrating them with minimal disruption to morphogenesis remains challenging. Here, we introduce a multiscale 3D microfluidic delivery platform that embeds lithographically defined, flexible, thread-like microchannels into organoids during growth. The nanoporous interface along the embedded microchannels enables controlled diffusive transport of biomolecules into localized regions with ~100 μm spatial resolution and to depths of ~400 μm from the organoid center, with minute-scale temporal precision, as demonstrated with dyes, morphogens, and MRI contrast agents. Delivery of growth factor–supplemented media leads to reduced apoptosis near the microchannels and improved neural tissue integrity. This platform offers a robust means to interrogate deep, site-specific microenvironments and to advance studies of organoid viability, structural organization, and functional maturation.

Recent advances in stem cell biology and tissue engineering enable the generation of neural organoids, which are three-dimensional (3D), self-organizing structures derived from pluripotent stem cells that mimic essential structural and functional aspects of the developing human nervous system. These models are valuable in studying neurodevelopmental disorders such as autism spectrum disorder, microcephaly, as well as rare monogenic conditions, and viral infections such as Zika and herpes simplex virus^1-3^, offering insights into human pathophysiological processes that cannot be reproduced in animal models due to differences in genetic backgrounds and disease manifestation. There is a growing interest in using organoids to study complex pathology and multisystemic interactions in aging and late-onset neurodegenerative diseases, like Alzheimer’s, amyotrophic lateral sclerosis and Parkinson’s disease^4-8^, as well as acquired conditions including traumatic^9-12^ and hypoxic injury^13^. Furthermore, organoid models serve as tools for more reliable and efficient drug screening and validation^14,15^.

Despite neural organoids providing more accurate and sophisticated models of human physiology than other *in vitro* techniques, they face critical limitations. Their internal structures lack continuous perfusion of cell culture media, resulting in reduced cell survival and restricted growth^16^. Studying these deeper layers is difficult due to the inability to provide base or differentiation media without disrupting the organoid’s intrinsic morphology. Another challenge arising from organoid self-organization is the shape heterogeneity across samples caused by the lack of physical constraints during growth under static conditions in multi-well plates. This variability hinders experimental reproducibility^17^, underscoring the need for complementary, integrated technologies to probe organoid structure, function, and dynamic behaviors in real time.

Current engineering strategies, such as organoid-on-a-chip technologies and culture chambers with porous polydimethylsiloxane (PDMS) membranes, attempt to recreate physiological environments to guide organization and improve base media delivery in peripheral layers of organoids. Bio-printed fluidic channels are widely developed for this purpose due to their biocompatibility and easy integration with spherical organoids^18^. The channels of previous technologies, however, scale at millimeters to a few hundred micrometers^19,20^. Such large features can alter the growth and morphology of the organoid tissue, limiting their application for comprehensive physiological studies. As a result, integrating microfluidic systems directly within intact organoids to deliver biomolecules deep into interior layers while preserving their intrinsic morphology has remained a major engineering challenge.

Here, we present a multiscale 3D microfluidic delivery platform that is naturally embedded into the tissue through neural organoid outgrowth. The device integrates microchannels with porous interfaces for targeted delivery of biomolecules to layers at depths of up to 400 μm in neural organoids while maintaining their intrinsic morphology and developmental trajectory. This approach provides a physiologically relevant fluidic microenvironment for organoid-based disease modeling and experimental neuroscience.

## Results

### Embedded 3D microfluidic architecture for intra-organoid delivery

**Extended Data Fig. 1 ∣:**
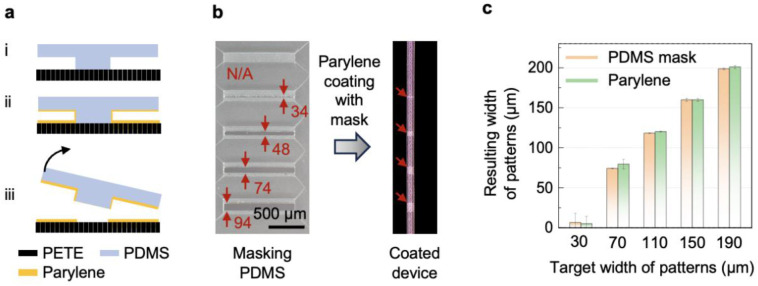
Lithography-based approach for selective parylene deposition. **a,** Schematic illustration of the PDMS masking process for selectively coating the parylene barrier. (i) The protruding structures of the PDMS mask adhere to the regions that will remain uncoated with parylene. (ii) The parylene coater deposits parylene uniformly across the entire surface of the sample. (iii) Removing the PDMS mask exposes the regions that remain uncoated with parylene. **b,** Optical images showing the dimensions of the protruding structures in the PDMS mask and the corresponding dimensions of the lithographically defined parylene-free regions. **c,** Comparison of the widths of the protruding structures in the PDMS mask and the resulting parylene-uncoated region.

**Fig. 1 ∣:**
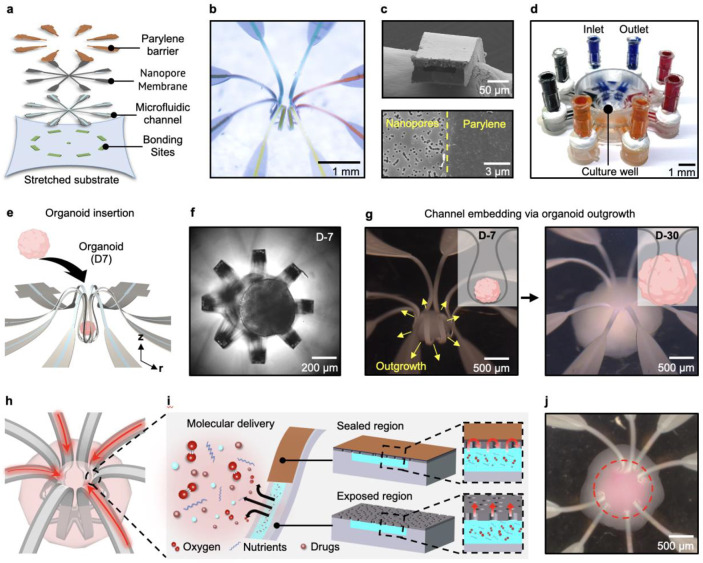
Design and implementation of multiscale 3D microfluidic platform for organoid interfaces. **a,** Schematic illustration of the layered microfluidic planar precursor before 3D transformation. **b,** Photograph of the microfluidic platform formed after transformation. **c,** SEM images of a cross-sectional side view of a microfluidic channel mounted on carbon tape (top), and top view of the boundary between the parylene-barrier-coated region of the nanoporous interface and the non-coated region (bottom). **d,** Photograph of the final configuration with the 3D-printed millifluidic module, including four pairs of inlets and outlets. **e,** Illustration of the insertion of a neural organoid into the approximately spherical cage formed by the buckled 3D microchannels. **f,** Top-view brightfield image focused on the organoid surface. **g,** Optical images of tissue outgrowth surrounding the channels after 20 days of differentiation. **h,** Schematic illustration of four-inlet delivery with flow directions indicated by red arrows. **i,** Schematic illustration of working principle of the site-specific molecular delivery through the nanoporous interface. **j,** Optical image of neuronal tracer diffusion toward the core of the organoid after 48 h of neuronal tracer delivery, as shown in dotted red circle.

Fig. 1 shows a representative example of a microfluidic-based interface for organoids.^21^ This type of multiscale, flexible, and high-resolution 3D microfluidic platform follows from geometric transformation of a planar precursor structure that consists of a lithographically defined collection of microchannels.(See Methods for details; Supplementary Fig. 1)^21,22^. In this case, the precursor consists of a collection of eight radially distributed microchannels extending from a central point (*r* = 0), formed using soft lithographic techniques in a polymer (Ostemer 324, Mercene Labs AB) (Fig. 1a).^23^ Specifically, the outer width of each microchannel expands from 60 μm at the central point (*r* = 0) to 2 mm at *r* = 11.7 mm, while the inner width for transporting fluids increases gradually from 20 to 200 μm over the same range (Supplementary Fig. 2). The total thickness of these structures (23 μm) and the depths of the microchannels (10 μm) are constant throughout each wing. A 10 μm-thick nanoporous membrane of polyethylene terephthalate (PETE; 200 nm pore diameter, 3 x 10^8^ ± 15% pores/cm^2^, Sterlitech) bonds on top of the microchannels, sealing them and forming an interface that enables biomolecules to diffuse into the adjacent tissue within the organoid. A 1 μm-thick layer of parylene coating on this membrane serves as a barrier coating on this membrane, with openings (*r* = 2.5 mm) lithographically defined on each microchannel to define sites where solutes within the channels can travel to the surrounding organoid (Extended Data Fig. 1). To achieve mechanical buckling, nine sites at the base of the precursor selectively bond the structure to a biaxially pre-stretched (41.6% strain) elastomer substrate (Ecoflex 00-45 Near Clear; Smooth-On, USA) using a silicone adhesive (Sil-PoxyTM, Smooth On). Upon releasing the pre-stretch, the precursor transforms via controlled buckling into a 3D architecture (Supplementary Fig. 3).

**Extended Data Fig. 2 ∣:**
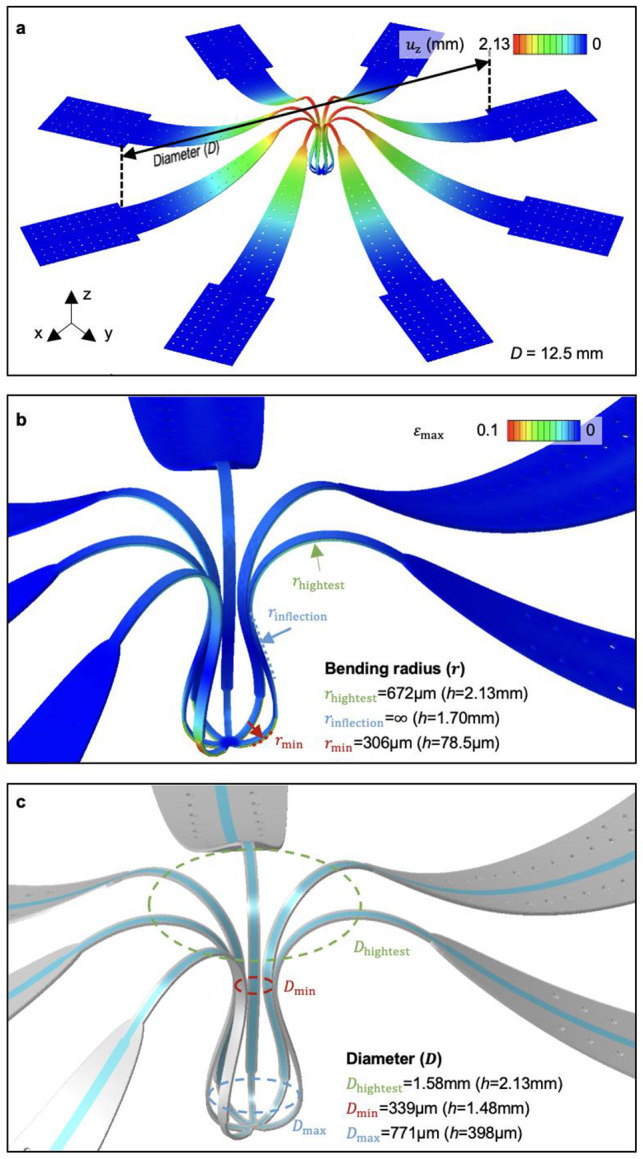
Dimensional features of the buckled structure predicted by FEA **a,** Overview of the buckled structure with a z-displacement color map. **b,** Maximum principal strain distribution and the bending radii across the continuously curved wing at different heights. **c,** The diameters of the cage structure in the x-y plane at the corresponding heights.

The resulting structure includes eight radially symmetric wings that emerge from the bonding sites in smooth, continuously curved geometries. Finite element analysis (FEA) and experimental observation were used to quantify the detailed 3D geometry of the structure (Fig. 1b and Extended Data Fig. 2). The central bonding site (*z* = 0), where the microchannels intersect, serves as the geometric center. From this point, the wings separate from the center axis (*r* = 0), forming a structure whose diameter increases gradually to 771 μm (at *z* = 398 μm) and subsequently decreases to 339 μm (at *z* = 1.48 mm), as the distance from the substrate surface increases. This configuration creates an approximately spherical, cage-like region designed to enclose a near-spherical organoid. Beyond this region, the bending orientation transitions to convex, with the diameter expanding to 1.58 mm at the highest point (z = 2.13 mm), before returning downward to the outer bonding sites (*z* = 0). Scanning electron microscopy (SEM) confirms the multiscale fluidic structure within each wing, showing a laminated nanoporous interface atop rectangular inner channels (60 μm × 10 μm) in a cross-sectional image (Fig. 1c, top). The boundary between sealed (parylene-coated) and exposed regions (nanoporous) on a single wing also confirms the distinctive surface characteristics that define the interface with organoid tissue (Fig. 1c, bottom). To connect with external tubing for controlled fluidic delivery, a 3D-printed millifluidic module is mounted to the periphery of the 3D microchannels (Fig. 1d). This millifludic module includes a ring (7 mm inner radius, 8 mm outer radius, and 3.4 mm depth) that bonds to the elastomer to define a well to contain cell culture media. Each adjacent wing pair functions as an upstream and downstream pathway, providing four independent delivery routes (Supplementary Fig. 4). The design achieves multiplexed operation, as illustrated by directing black, red, blue, and yellow dyes through the four pairs of wings (Fig. 1d).

**Extended Data Fig. 3 ∣:**
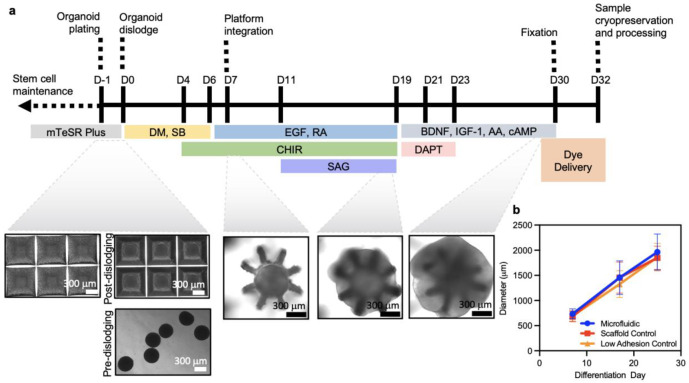
Experimental timeline and organoid diameter tracking. **a,** Morphogen delivery and experimental timeline with representative brightfield images. **b,** Diameter measurements, with means and standard deviations, through differentiation protocol in microfluidic, scaffold control, and low adhesion control samples.

The 3D architecture of the microfluidic platform enables integration into the inner region of an organoid that is grown within the cage. Experiments to demonstrate this process begin with the separate preparation of spinal cord organoids grown for seven days in low-adhesion plates to diameters of ~550–580 μm (Extended Fig. 3a). Deforming the elastomeric substrate temporarily enlarges the circular aperture (~339 μm in diameter) located at the top-center region of the structure (Fig. 1e). This expansion enables insertion of an organoid into the underlying cage (diameter ~630 μm) and allows the organoid to remain in place after the substrate returns to its undeformed state (Fig. 1f). Under continuous media refreshment in the well, growth of the organoid leads to formation of tissue structures that protrude outward through the gaps between the wings. After 20 days, this process leads naturally to a complete embedding of the 3D microchannel structures within the organoid (Fig. 1g). The diameter of the organoid reaches an average of 1.9 ± 0.2 mm at day 25 (Extended Data Fig. 3b).

The fully embedded configuration enables targeted biomolecular intraorganoid delivery through the open nanoporous interfaces in each wing, as shown in Fig. 1h. In this case, the parylene-coated region of each wing extends from the circular aperture at z = 1.48 mm to the periphery of the 3D microchannels, while all other regions at the cage remain exposed. This site-specific sealing allows targeted delivery and prevents solutes within the microchannels from leaking directly into the external medium in the culture well (Fig. 1h). Flowing a neuronal tracer (Cholera Toxin Subunit B, Alexa Fluor 555 Conjugate, Invitrogen) enables visualization of the delivery function. After 48 h incubation, the tracer permeates the organoid tissue, appearing as a red signal extending confined primarily to the intra-volume of the organoid (Fig. 1j).

### Intraorganoid Delivery Dynamics

**Extended Data Fig. 4 ∣:**
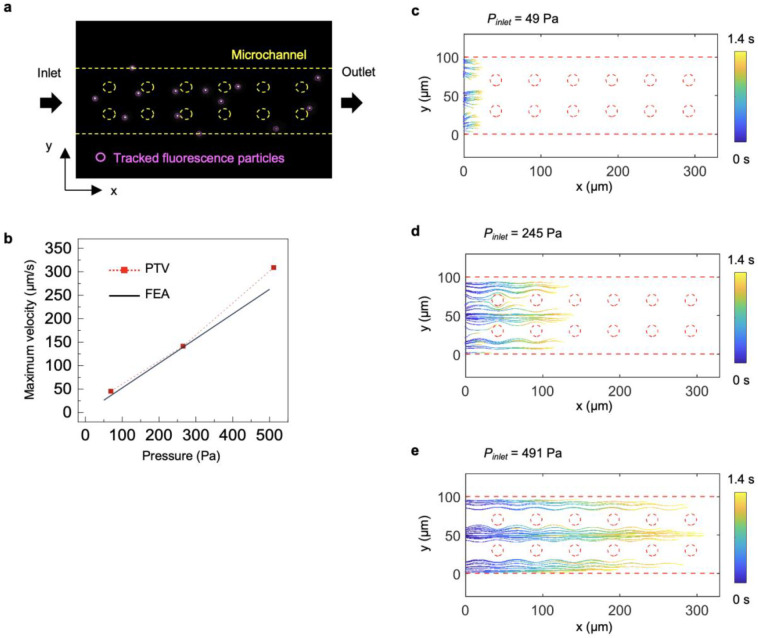
Particle tracking velocimetry (PTV) for visualizing fluid flow in the microfluidic platform. **a,** Optical microscopy image showing the flow of a microparticle suspension in a wing microchannel for performing PTV. **b,** Comparison graph between FEA results and experimental measurements of the velocity of pressure-driven flow at different applied pressures. **c-e,** PTV analysis in the microchannel over 1.4 s at the applied pressures, performed to calculate the maximum velocities shown in the comparison graph.

**Extended Data Fig. 5 ∣:**
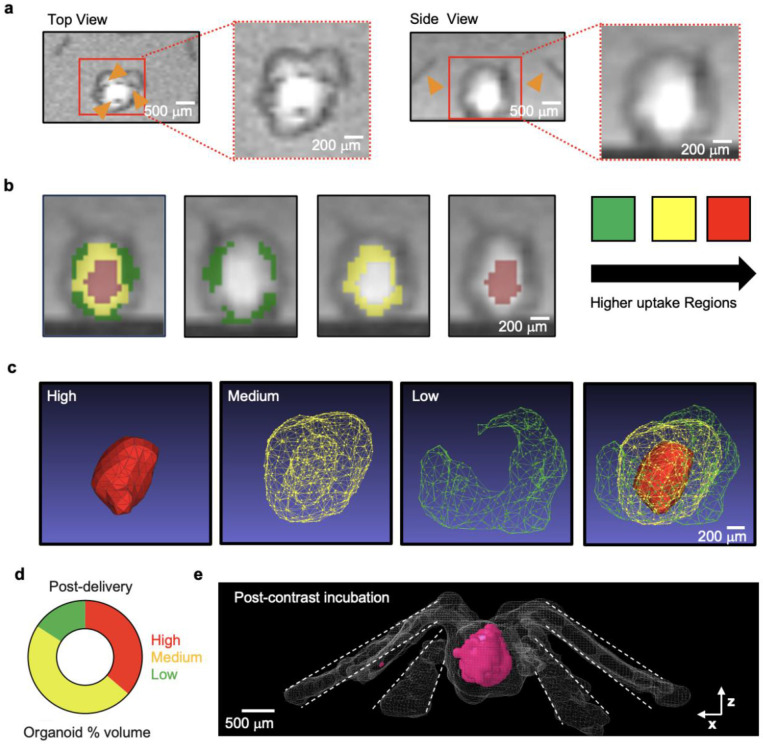
MRI intensity segmentation for Gadolinium delivery quantification. **a,** Representative post gadolinium incubation 2D MRI images of microfluidic organoid shown from top and side-view. The microfluidic channels and legs appear as hypointense structures (indicated by orange arrows). The enlarged images corresponding to each orientation enable visualization of the heterogeneous uptake of contrast delivered via the different channels. **b,** Representative 2D side-view MR image shows superimposed colored ROI corresponding to portions of the organoid with different uptake of MRI contrast as delivered through the microfluidic channels. These three different ROIs were selected in 3D using the MRI intensity as marker of contrast uptake. The selected threshold approach used three different windows (color coded as red for high uptake, yellow for intermediate uptake and green low uptake). **c,** MRI-segmented layers of a single organoid due to differences in diffusion and **d,** quantified compared to baseline diffusion. The layers are divided into three intensities groups marked in red, yellow, and green. **e,** 3D reconstructed image of 3D fluidic channels embedded in an organoid lighted in pink. Channel wings are traced in white dotted lines.

**Extended Data Fig. 6 ∣:**
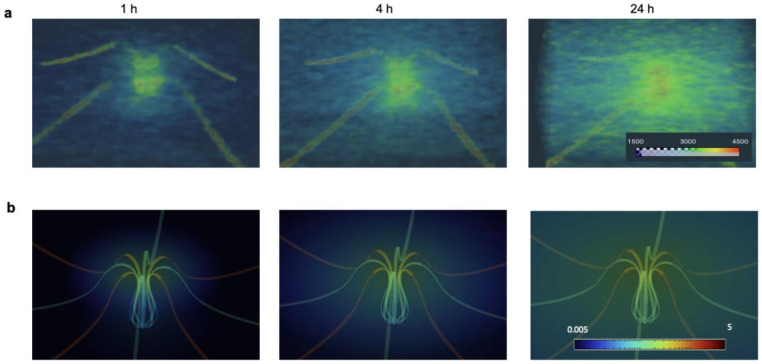
Selected time-lapses of iohexol (300 mg/mL) diffusion through a 3D fluidic device embedded in 1% agarose with micro-CT imaging. **a,** 3D reconstructed images after 1, 4, 24 h of delivery and **b,** respective FEA simulations. The inlet pressure for delivery is 196 Pa.

**Fig. 2 ∣:**
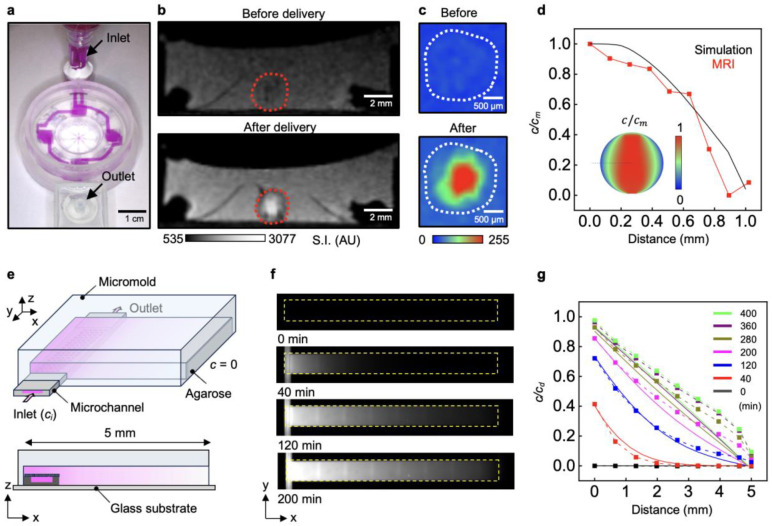
Investigation of intra-organoid solute delivery through the porous interface in the 3D microfluidic platform. **a,** Photograph of a 3D-printed millifluidic module delivering reagent simultaneously through all wings. **b,** MRI images acquired before and after delivery of a contrast agent through the platform, with gray-scale signal intensity indicating agent concentration. **c,** Magnified view of the organoid region from MRI images with a color map. The white dotted contour outlines the embedded organoid. **d,** Line plot of normalized concentration distributions along the radial direction from the organoid center, comparing FEA simulations with experimental measurements, *c*_*m*_=5.6 mM. **e,** Schematic illustration of the experimental setup for time-lapse optical microscopy of mass transfer across the nanoporous interface into an agarose phantom. **f,** Time-lapse images showing diffusion of fluorescent molecules from a single wing into agarose. The yellow box has dimensions of 5 mm (x-axis) and 500 μm (y-axis). **g,** Transient normalized concentration distributions of fluorescent dye along the x-axis obtained from time-lapse fluorescence microscopy, *c*_*d*_=10 μM. The dotted lines with symbols represent the experimental results and solid lines correspond to the FEA results.

A key aspect of intraorganoid delivery is mimicking the multiscale, networked fluidic paths of the vascular system, which supports both advective fluid transport and diffusive solute exchange. Two-dimensional (2D) and 3D visualization techniques together with FEA models for a microchannel-embedded organoid allow investigation of essential transport mechanisms, focusing specifically on diffusive solute exchange. In the 3D magnetic resonance imaging (MRI) setup (Fig. 2a), a 5 mM gadolinium contrast reagent (Magnevist, Bayer, Germany) enters the inlet branches and then into the largest dimensions of the microchannels with impermeable walls. This process enables uniform fluid distribution and favors advective transport with reduced hydraulic resistance, even under low hydrostatic pressure (690 Pa) without leakage (Extended Data Fig. 4). Furthermore, this experiment demonstrates that the microfluidic wings exhibit localized permeability, enabling bidirectional diffusive exchange of solutes between the microchannels and the embedded organoid. After delivering the contrast reagent for 1 h (Fig. 2b), cross-sectional slices of an MRI indicate that the maximum signal intensity occurs in the inner regions of the organoid (Fig. 2c and Extended Data Fig. 5), confirming that reagent transport proceeds from the nanoporous interface into the organoid and subsequently toward the culture well in 3D. Results from computed tomography (CT) imaging confirm this trend (Extended Data Fig. 6). A line plot of the signal intensity reveals a radial solute gradient with a gradual increase in slope from the center toward the surface, implying diffusion-dominated transport characteristics (Fig. 2d). The advection–diffusion model for porous media yields an effective diffusivity of the contrast agent of ~40 μm^2^/s through the nanoporous interface, a Peclet number of 0.015, and a maximum radial advection velocity of ~0.55 μm/s within the organoid at 0.21 mm from the center, without any parameter fitting, serving as a guideline for theoretical estimation of other molecular concentration distributions within organoids.

**Extended Data Fig. 7 ∣:**
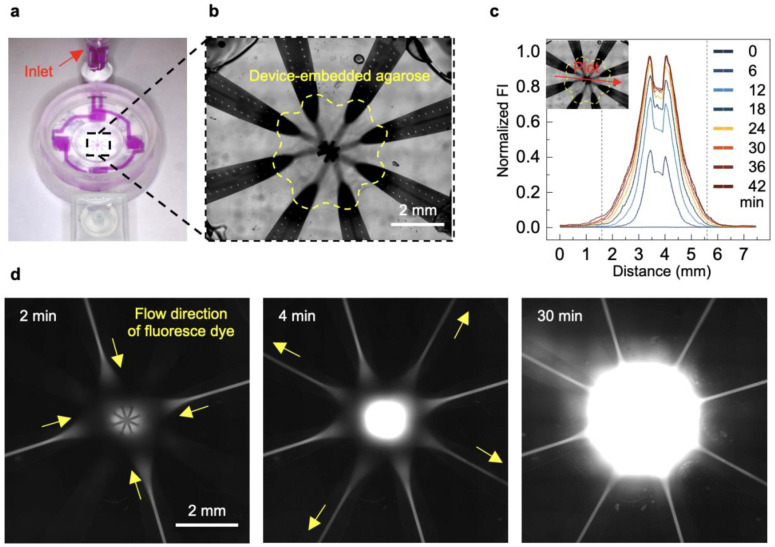
Optical microscopy of fluorescent-molecule delivery through the porous interface into a phantom model. **a,** Photograph of a 3D-printed millifluidic module used to deliver dye simultaneously into all the wings for microscopy. **b,** Microscopic view of the microfluidic platform, where the porous interface lies embedded within 1% agarose. **c,** Line plot showing changes in fluorescence intensity (FI) over 42 min along the line indicated by the red arrow, when 10 μM SrB fluorescent dye flows through the microchannels. **d,** Optical fluorescence microscopy images at different time points showing the SrB delivery corresponding to the line plot.

The experimental setup for a 2D diffusion model study employs time-lapse microscopy with shortened acquisition times and higher-resolution imaging to resolve transient solute-transport dynamics in a wing with improved temporal precision. In this system (Fig. 2e and Supplementary Fig. 5), a micromold made of Ostemer forms a 1% agarose organoid phantom with a predefined geometry that includes a sink region at one end, wherein the solute concentration remains fixed at zero, while the remaining boundaries serve as closed conditions. The micromold also positions a single microfluidic wing at a defined location to impose a well-controlled source concentration boundary within the agarose. Dye delivery involves passage of 10 μM fluorescent sulforhodamine B (SrB) through the wing, leading to diffusion across the nanoporous interface and into the agarose along the x-axis (Fig. 2f). Line profiles extracted from fluorescence images acquired across 400 min at 40-min intervals visualize the transient diffusion between the source and sink, which is in good agreement with FEA simulations (Fig. 2g). The 5 mm-long agarose phantom reaches steady state in under 6 h, demonstrating an efficient average solute flux of 1.8 μM·μm·s^−1^ across the nanoporous interface. 3D microchannels embedded with 1% agarose also demonstrate transient diffusion, achieving minute-scale temporal precision (Extended Data Fig. 7).

### Delivery of morphogens, cellular and neuronal dyes

**Extended Data Fig. 8 ∣:**
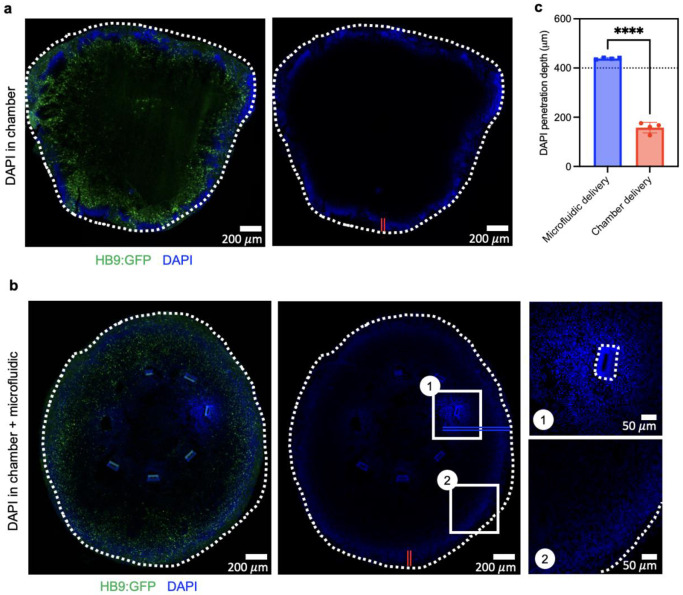
DAPI organoid penetration in organoids. **a,** Immunofluorescence images of low adhesion organoid with DAPI delivered in chamber and **b,** with DAPI delivered through the microfluidic and chamber. ROI 1 shows DAPI delivery in near the channel, and ROI 2 shows passive diffusion through the chamber. Double red line shows penetration depth from chamber diffusion, and double blue line shows penetration depth acquired through microfluidic delivery. **c,** Bar graph showing increased penetration depth acquired with microfluidic delivery, with dotted line showing average position of microfluidic channel. (n= 4 organoid sections/condition)

**Fig. 3 ∣:**
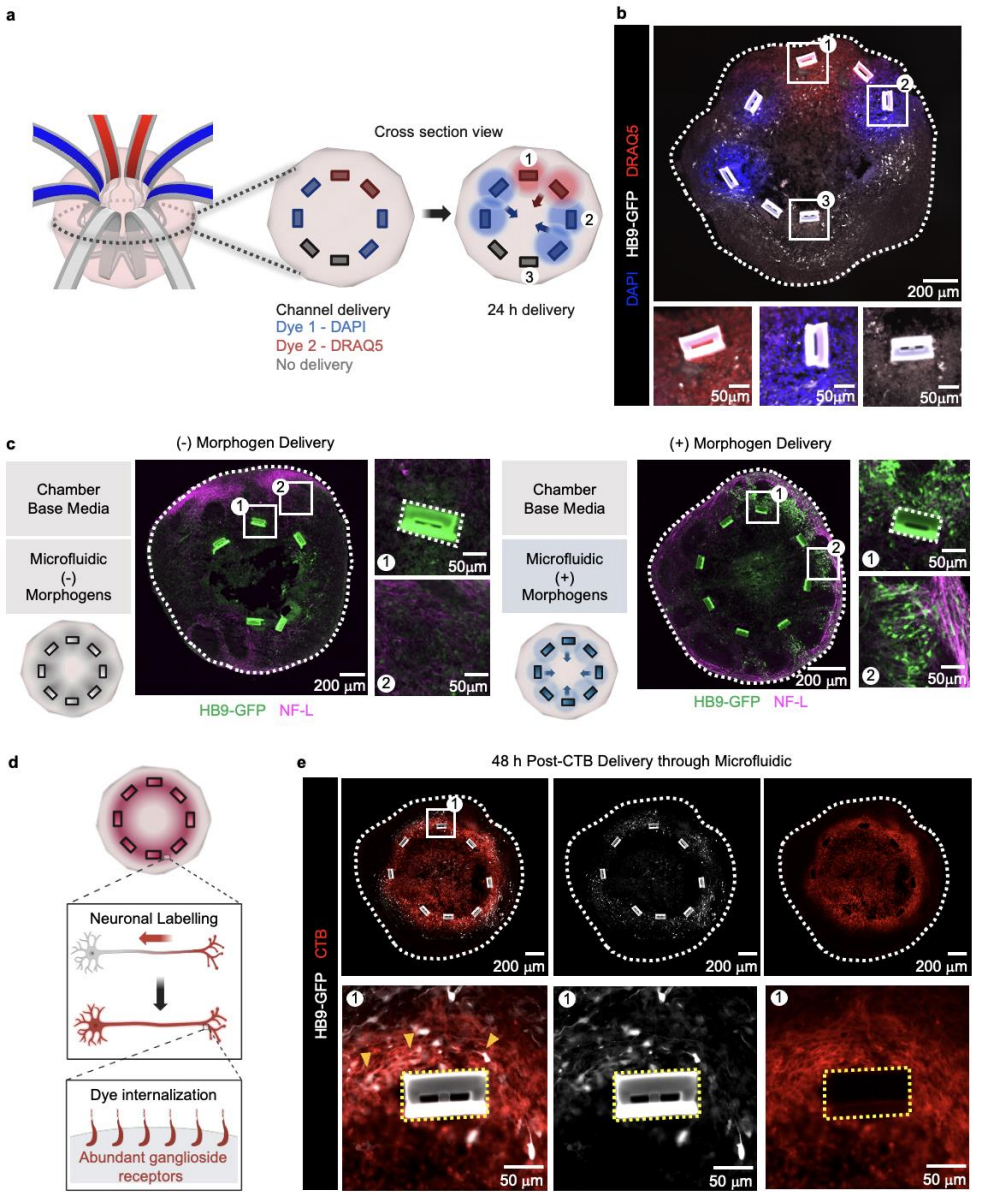
Fluidic platform delivery of nuclear dyes, morphogens, and neuronal tracer to organoid. **a,** 3D schematic illustration of alternating dye delivery with cross-sectional view indicating nuclear dyes and control (no delivery) following expected 24-hour incubation delivery. **b,** Organoid cryosection validates successful alternating dye delivery, with three distinct ROIs near microfluidic channels. ROIs 1-3 each show DRAQ5 (red), DAPI (blue), and no delivery, respectively. HB9-GFP motoneuron and microfluidic channel autofluorescence are adjusted as white. **c,** Targeted delivery using motoneuron differentiation-specific morphogens. Whole cross-section, ROI 1 near channel, and ROI 2 in periphery for organoid with basal media in chamber and microchannels (left), and basal media in chamber and morphogens delivered through microchannels (right). NF-L shown in pink, and HB9-GFP motoneuron with autofluorescence channels shown in green. **d,** Schematic illustrating CTB-555 selective labeling through a single neuron. **e,** Representative organoid section image with CTB-555 at day 30, followed by ROI near fluidic device in dotted yellow square with sample positive HB9 and CTB cells marked with orange arrows.

To further characterize the ability of the microfluidic system for multiplexed delivery of biomolecules in the tissue microenvironment near the organoid interface, standard DNA-binding dyes DAPI and DRAQ5 are delivered through the microfluidic wings after 30 days of differentiation. The passive diffusion of nuclear dyes through cell membranes in cryosections after 24 h shows fluidic channel delivery accessing greater penetration depth (up to 438.5 ± 3.2 μm) compared to the typical value (160 ± 21.5 μm) achieved by passive diffusion from cell maintenance media in the chamber external to the organoid (p< 0.0001; Fig. 3a, Extended Data Fig. 8). Consistent with MRI findings, imaging reveals targeted nuclear uptake in cells near the microfluidic wings embedded within organoids (Fig. 3b). Alternating multiplexed dye staining is achieved through separated pairs of microchannels, where each wing can deliver different nuclear dyes (Fig. 3b, ROI 1-2). Absence of dye in a fluidic channel pair is used as a negative control (Fig. 3b, ROI 3).

Targeted molecule delivery provides a means to investigate the use of an embedded 3D microchannel structure for spatially directed differentiation within a single organoid. For this purpose, specific morphogens like smoothened agonist (SAG, 1 μM); 566660, Millipore) and retinoic acid (0.1 μM; R2625-50MG, Sigma Aldrich), which promote the differentiation of ventral spinal cord cell types including motoneurons in organoids derived from human embryonic stem cells (hESCs), were selectively passed through the 3D microchannel structures. A negative control group receives base media without morphogens. Subsequent immunohistochemistry demonstrates successful differentiation of HB9-GFP+ motoneurons in the organoid sections receiving targeted delivery of ventralizing morphogens via embedded 3D microchannels. In comparison, organoids only receiving basal media develop neurofilament positive networks with sparse HB9-GFP+ motoneurons. Overall, these results indicate successful spatially-specific differentiation of hESCs into spinal motoneurons in organoids and further demonstrate the functionality of the embedded microfluidic system in directing localized morphogen delivery (Fig. 3c).

To further test neuronal specific dye delivery via our platform in live organoids, cholera toxin subunit B-555 (CTB-555) is introduced directly through fluidic microchannels. CTB is a widely used neuronal label in neuroscience experiments, as it binds GM1 gangliosides on neuronal plasma membranes and becomes internalized via endocytosis, labelling both axon and soma (Fig. 3d)^24^. Delivery through microchannels enables CTB-555 penetration into deeper regions of the organoid, where labeled neurons can be visualized in proximity to microchannels (Fig. 3e). Importantly, optical imaging prior to fixation and cryopreservation confirms targeted delivery towards the core of the organoid, highlighting the capacity of the device to achieve spatially controlled neuronal labeling (Fig. 1j).

### Structural changes in spinal cord organoids with embedded microchannels

**Fig. 4 ∣:**
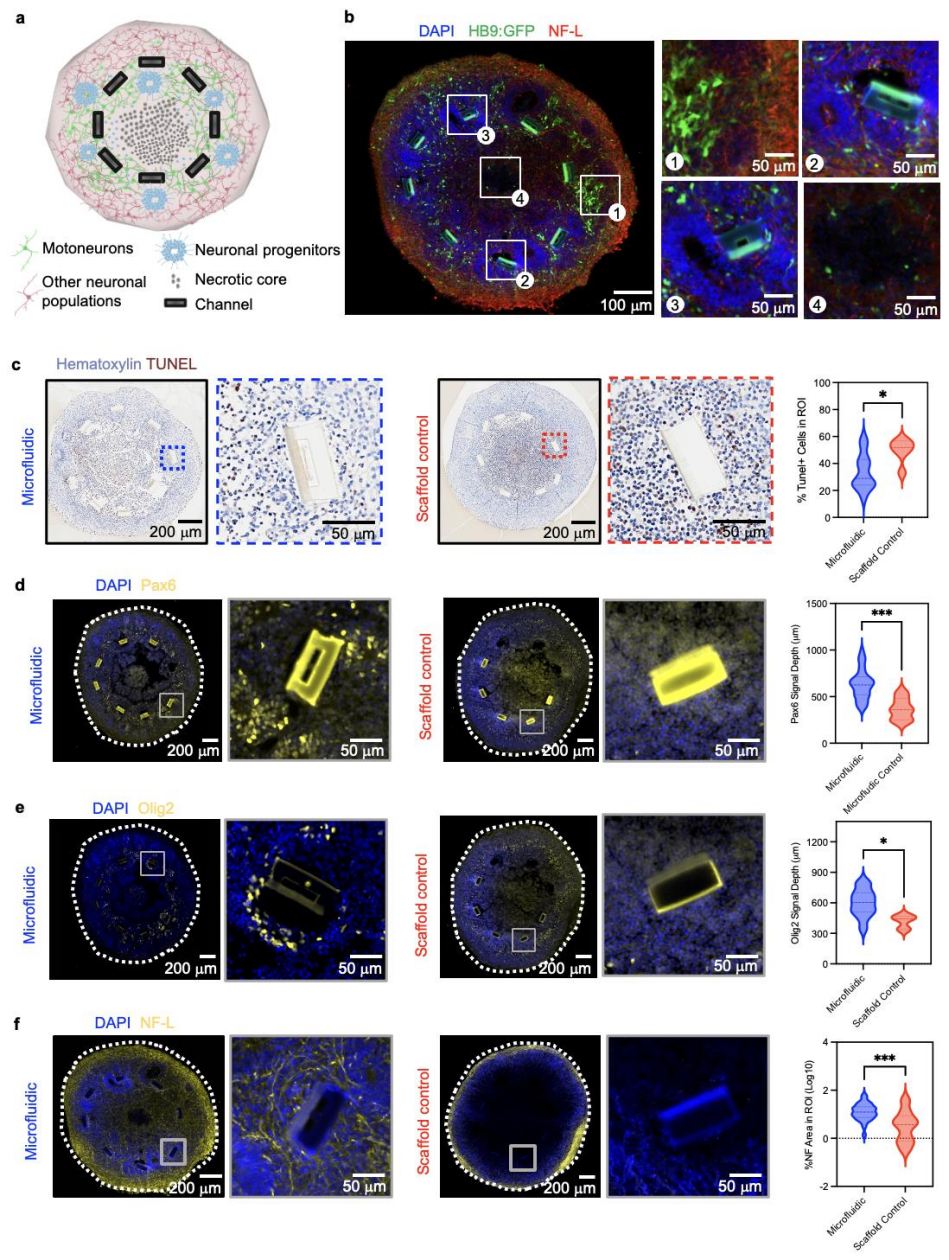
Structural characterization of integrated microfluidic organoid interface. **a**, Cross-sectional schematic of spinal cord organoid structural morphogenesis embedded with fluidic microchannels. **b,** Immunocytochemistry in a cryosection of an organoid with a fluidic device on day 30. ROIs 1-2, 3, and 4 show the microenvironments near the microfluidic channel, at the necrotic organoid core, and at the organoid periphery respectively. **c,** Paraffin sections of TUNEL assay for apoptosis quantification in ROIs near the microchannels, and quantification of % TUNEL+ cell in ROIs (n = 12 microfluidic, 6 scaffold control). Representative immunofluorescence images of organoid cryosections with markers of interest **d,** PAX6, **e,** OLIG2 and **f,** NF-L in microfluidic and control samples. Violin plots show quantification of signal depth of PAX6 and OLIG 2 (n = 12 microfluidic, 3 control) and percentage area of NF-L (n = 30 microfluidic, 16 scaffold control). * p < 0.05; **p < 0.01; ***p < 0.001; ****p < 0.0001).

Additional experiments evaluate the capacity to leverage the platform to study and modify specific neural organoid microenvironments. A proof-of-concept protocol applies the platform to spinal cord organoids in a design that is compatible with diverse organoid systems. Spinal cord organoid morphogenesis consists of segregated layers of specific cell types (Fig. 4a). As observed in region of interest (ROI) 1, the outermost layers are composed of diverse neuronal populations, including motoneurons (Fig. 4b). The intermediate layer prominently consists of neuronal progenitor cells and rosette structures, where the 3D microchannels reside. This configuration is evident in ROIs 2 and 3 of Figure 4b, where rosettes form within proximity to the channel structures. The innermost layer, by contrast, contains apoptotic cells (Fig. 4a) and no neuronal cells due to the lack of perfusion (Fig. 4b, ROI 4). To assess whether fluidic delivery significantly improves cell survival in these deeper regions, the proportion of apoptotic cells (TUNEL+) in ROIs adjacent to microchannels delivering differentiation media are compared to that of scaffold controls (Fig. 4c). ROIs near microchannels exhibit a significantly lower percentage of TUNEL+ cells (median 28.9%, IQR 23.9- 43.2) compared to scaffold controls (median 51.9%, IQR 43.9-56.7; p= 0.0179), representing a nearly two-fold decrease (Fig. 4d). These results indicate that the device creates a localized sustaining effect, enhancing viability and reducing apoptosis in deeper layers of the organoid embedded by 3D microchannels.

**Extended Data Fig. 9 ∣:**
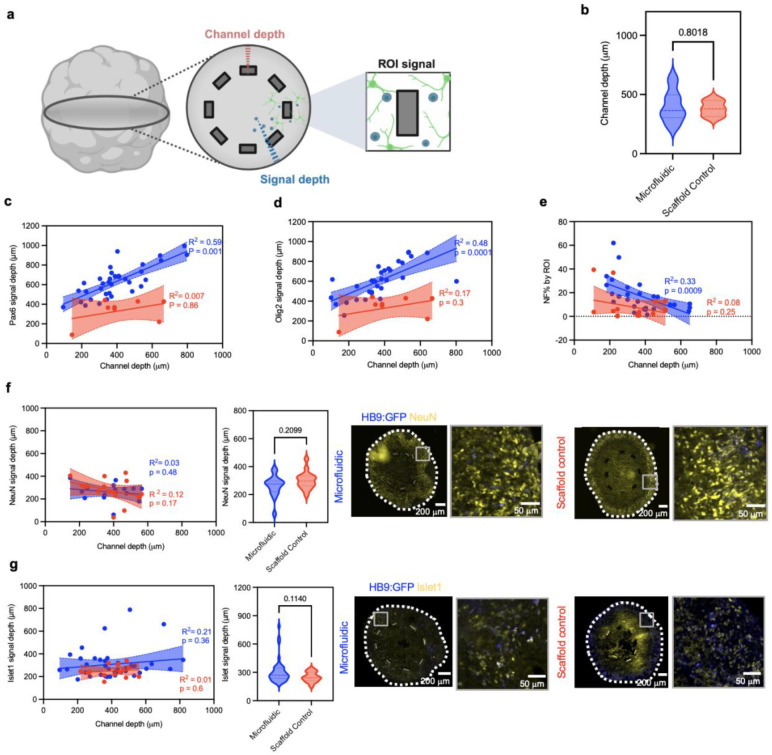
Channel depth and extended characterization of neuronal progenitors and post-mitotic neuronal markers. **a,** Schematic of analysis for: channel depth (distance from the organoid surface to fluidic channel), signal depth (distance from organoid surface to deepest signal within organoid), and quantified signal depth within ROI adjacent to fluidic channel. **b,** Channel depth quantification in microfluidic and scaffold control shows no difference in position within organoid (n = 12 microfluidic, 3 scaffold control). Scatter plots show correlation between channel depth and marker signal depth for **c,** PAX6 and **d,** OLIG2 (n= 12 microfluidic, 3 control, each dot represents one wing of a sample), and **e,** %NF in ROI for NF-L, showing R^2^ and p values, and error bands representing 95% confidence intervals (supplemental to figure 4). Scatter plots for **f,** NeuN and **g,** Islet1 (n = 16 microfluidic, 16 control, each dot represents one wing of a sample), followed by Violin plots show no difference in signal depth of each marker between microfluidics and scaffold controls. Representative immunofluorescence images of organoid cryosections in microfluidic and control samples showing peripheral prominence in post-mitotic neuronal markers (****p < 0.0001).

Utilizing the same experimental set-up, the spatial distribution of neuronal progenitor associated transcription factors can be quantified. The focus is on expression of PAX6 and OLIG2, which mark distinct progenitor domains within the developing spinal cord and play complementary roles in progenitor patterning, cell cycle regulation, and specification of ventral lineages including spinal motoneurons^22^. Measurement of signal depth (distance from the organoid border towards the core) reveals that PAX6 appears at depths of up to 633.2 ± 158.9 μm in samples with microfluidic delivery compared to scaffold controls (370 ±120 μm; p< 0.0004) (Fig. 4d, Extended Data Fig. 9c). Similarly, OLIG2 expression extends deeper (600 ± 135 μm) in samples with microfluidic delivery compared to scaffold controls (420 ± 70 μm; p = 0.0457) (Fig. 4f, Extended Data Fig. 9d). Additionally, quantification of expression area of neurofilament light chain (NF-L), a key component of neuronal cytoskeleton, shows an average of 12% more coverage in ROIs adjacent to microfluidic delivery (IQR - 7.4-20.54) compared to scaffold controls (median 3.7 %, IQR 0.76 - 9.4; p = 0.0008) (Fig. 4g, Extended Data Fig. 9e). In contrast, markers of postmitotic neurons have more superficial localization (Fig 4a). ISLET1, a transcription factor essential for spinal motoneuron maturation, is predominantly found closer to the organoid border in microfluidic delivery samples (median 269.8 μm, IQR 236.0- 347.0) relative to scaffold controls (median 243.3 μm, IQR 211.4- 303.5, p = 0.11). NeuN+ cells, a neuronal nuclear protein widely used as a marker for mature neurons, show a median 297 μm signal depth in microfluidic delivery samples (IQR 261.1- 334.4) compared to that of scaffold controls (median 275 μm, IQR 220.2-297.0, p = 0.209) (Extended Data Fig. 9f-g). In summary, these data indicate that microfluidic delivery preferentially enhances penetration and distribution of progenitor and neurogenic markers in deeper regions of the organoid, while markers of mature neurons remain largely confined to superficial layers, where access to nutrients and oxygen from the external culture medium is likely sufficient.

## Discussion

This work introduces a 3D multiscale microfluidic platform that can be naturally integrated with neural organoids while delivering base media, morphogens, and fluorescent dyes to previously inaccessible inner organoid layers. Immunostaining results demonstrate decreased apoptotic cells near the microfluidic channels and increased NF-L percent area, suggesting successful nutrient delivery through the microchannels. Targeted delivery of CTB-555, a neuronal specific dye, and general nuclear dyes, such as DAPI and DRAQ5, confirm extended penetration depths up to three times higher than those from passive diffusion of media surrounding the organoid. Accessing larger distances and enhancing diffusion is an important step towards facilitating more stable and consistent long-term cultures to study mechanisms underlying age-related disorders.

Beyond molecular delivery, this system enables applications of contrast delivery and non-destructive, longitudinal MRI acquisition. Because each fluidic wing can be independently controlled, this platform supports multiplex experiments within a single organoid, such as parallel drug screening to facilitate insights into treatment development for neurodegenerative, neuro-oncology, and neurotrauma, among other conditions. Additionally, targeted spatiotemporal delivery of differentiation cues may enable the formation of morphogen gradients that are known to drive regionalization during central nervous system development^25^, providing a framework for spatial organization to model organ development such as in polarized brain organoids^26^. Together, these results demonstrate targeted delivery of dyes, morphogens, and MRI contrast agents and improved access to deeper organoid regions that are difficult to reach by diffusion alone. The modular platform design enables straightforward extension to additional delivery modalities.

Through targeted cell nutrient delivery, this soft, flexible device has strong potential as a next-generation tool for improving organoid health and non-destructive access to organoid interior for experimentation. However, complete resolution of central necrosis remains a challenge, highlighting an important direction for future optimization. Further iterations of this technology can be focused on modifications in spatial designs and choices of materials. Specifically, the exposed surface areas of the nanoporous regions on the 3D microchannels can be increased to improve spatial diffusivity and even potentially serve as vasculature pathways, while gas-permeable materials can be utilized for the microchannels to supplement fluidic perfusion into soft biological tissues. Other enhancements to the platform may include additional modalities such as microelectrodes to study functional synaptic connectivity within deeper organoid structures. Altogether, the application of this technology can facilitate long-term cultures and spatiotemporal control of differentiation factors, ultimately enabling more physiologically relevant models for neural development and disease modeling.

## Methods

### Fabrication of 3D microfluidic architectures

Fabrication of the 3D microfluidic platform involved five main steps: soft lithography, lamination, deposition, etching, and packaging (Supplementary Fig. 1). The first step formed the microchannel layer in Ostemer (Ostemer 324; Mercene Labs AB). The process began with spin-coating polyvinyl alcohol (PVA; 2 wt% in DI water, 1000 rpm, ~53 nm thick) onto a pre-cleaned glass slide to form a sacrificial lift-off layer. Exposing the PVA surface to an oxygen-plasma treatment (March RIE; 10 sccm O_2_, 50 W, 220 mTorr, 15 s) and to vapor-phase (3-glycidyloxypropyl) trimethoxysilane (GPTMS) for 30 min increased the surface energy. A PDMS mold placed on the treated surface and degassed for 1 h enabled spontaneous infiltration of Ostemer resin into the gap between the mold and the PVA-coated substrate. Curing the resin under UV-induced ozone for 10 min allowed the microchannel layer to be removed from the mold. The lamination step involved bonding a PETE membrane to this layer to form a sealed microchannel structure. The process began with oxygen-plasma treatment of the PETE membrane (10 sccm O_2_, 100 W, 220 mTorr, 1 min), followed by vapor-phase GPTMS exposure for 1 h. After uniform lamination of the surface-treated PETE onto the lithographically defined microchannel layer, the laminated assembly was baked at 80 °C for 24 h to promote covalent bonding between the surfaces. In the deposition step, a thin parylene layer was selectively coated onto the laminated assembly using a soft-lithography–assisted masking approach. The process began by placing a microfabricated PDMS mask onto the PETE surface. A 1 μm-thick parylene layer was then deposited using a parylene coater (SCS Labcoater 2; Specialty Coating Systems Inc., USA). Removal of the PDMS mask produced a patterned parylene layer along the mask-defined boundary (Extended Data Fig. 1a). The parylene-coated device was subsequently lifted off the substrate by dissolving the PVA sacrificial layer. In the etching step, the PETE sheet was patterned into the same 2D layout as the microchannel layer. The parylene-coated device was placed onto a thermal release tape (tape 1; 3198M, Semiconductor Equipment Corp.) with the Ostemer side facing upward. The exposed PETE surface was etched using reactive-ion etching (March RIE; 25 sccm O_2_, 220 W, 220 mTorr, 50 min), while the Ostemer microchannel layer protected the underlying regions of the PETE. The etched device was transferred to a single-sided PVA tape by thermally releasing tape 1 at 130 °C. This intermediate PVA tape was then laminated onto a second thermal release tape (tape 2; RA98LS, Semiconductor Equipment Corp.), after which the PVA tape was dissolved, leaving the etched device securely attached to tape 2 for subsequent processing.

### Assembly of multiscale 3D fluidic platform

The 3D assembly process began with pre-stretching a elastomer substrate (1.3 mm thickness, in a square shape with side length 12 cm) made of a low-modulus silicone material (Ecoflex 00-45 Near Clear, mix ratio 1:1; Smooth-On, USA) to a biaxial strain of 41.6% by securing it onto a double-axis stage as described in a previous method.^21^ A silicone epoxy adhesive (Sil-Poxy^™^, Smooth-On) applied through a polyimide mask (140 μm-thick with eight 1.5 mm × 2.5 mm rectangular openings) defined one bonding site on each wing and a circular bonding region (110 μm-diameter) at the center. Contacting the structure against the stretched elastomer substrate and curing for 1 h at room temperature led to strong bonding at these sites. Baking at 120 °C released tape 2, to allow the geometrical transformation process to proceed upon releasing the prestrain to 8.0% based on FEA results to create the appropriate size for 3D channel structure without each wing touching/overlapping with each other. Specifically, compressive forces at the bonding sites convert the 2D structure into a 3D framework. A laser-cut ring-shaped PMMA substrate (35 mm outer diameter, 15 mm inner diameter, 1 mm thickness) adhered to the backside of the elastomer substrate with Sil-poxy provided mechanical reinforcement. The bottom of this elastomer will later be pushed in an upward motion to manually open the 3D framework for organoid insertion. Regions of the square elastomer that extended beyond the circular PMMA substrate were cut away. Finally, the cut-out structure is adhered with Sil-poxy to a 3D-printed millifluidic module in alignment to the periphery of the buckled 3D microchannel.

### FEA simulations

The nonlinear post-buckling mechanical behavior of 3D structures under compression was analyzed and predicted using FEA with the commercial software Abaqus. Eight-node solid elements were selected to model the precursor structures, and mesh convergence tests were conducted to ensure computational accuracy. A linear buckling analysis of the 2D precursor structures under compression was performed to determine the critical buckling strain and corresponding buckling mode. These results were then incorporated as initial geometric imperfections in the post-buckling simulations. The deformed configurations and strain distributions of the 3D structures were obtained at various pre-strain levels. The elastomeric materials exhibiting hyperelastic behavior were modeled using the second-order incompressible Yeoh hyperelastic constitutive model. The material properties used included OSTEMER with an elastic modulus of *E*_OSTEMER_ = 10.29 MPa and PETE with an elastic modulus of *E*_PETE_ = 2 GPa.

Numerical simulations of diffusion through bucked structures were conducted using COMSOL Multiphysics^™^, employing tetrahedral mesh elements for all computational analyses. A refined mesh with feature sizes smaller than 1/6 of the channel width was adopted to ensure accuracy. The geometry of the buckled structure was exported from Abaqus using the 3D mesh-to-geometry plug-in and imported into COMSOL via the CAD Import Module add-on. An oblate spheroid organoid (semi-axes *r*_0_ = 1.0 mm, *r*_0_*_z_* = 0.98 mm), based on experimental measurements, was positioned such that its bottom plane aligned with that of the buckled structure. Fluid flow was modeled using the Creeping Flow interface with porous media domains, appropriate for the low Reynolds numbers involved (Re = 0.0084 for Magnevist delivery; Re = 0.0032 for Iohexol delivery). Diffusion behavior was simulated using the Transport of Diluted Species in Porous Media module, incorporating convection coupled with fluid flow. The effective diffusivity model, assuming a tortuosity factor of 1, was applied within the porous media domains. The numerical solver dynamically controlled time-step sizes through backward differentiation formulas (BDFs), with initial step sizes kept small to prevent singularity. The boundary conditions were set to no-slip, with specific configurations at the inlet and outlets. The inlet end was set to a fully developed flow under pressure of 690 Pa (hydrostatic) for Magnevist delivery, 510 Pa for fluorescent dye SrB delivery, and 196 Pa for Iohexol delivery. The outlet pressure was maintained at 0. Water was modeled as Newtonian fluids with the following parameters: *ρ*_w_ = 997 kg/m^3^, *η*_w_ = 0. 0010016 Pa·s for liquid water. In the 2D diffusion model, the PETE membrane was modeled as unsaturated medium with effective diffusivity given by *D*_eff_ = *sε*_PETE_*D*_dye_ , where *s* was the saturation, assumed to be *s* = *s*_0_ + *k*_*s*_*t*. *t* was time, *k*_*s*_ was the rate of change of saturation over time, and *s*_0_ was the initial saturation. Fitting parameters were initial saturation *s*_0_ being 0.075, and the rate of change of saturation *k*_*s*_ being 3.7E-5 s^−1^. Diffusivity values in water were: Magnevist *D*_m_ = 450 μm^2^/s^27^, fluorescent dye SrB *D*_dye_ = 470 μm^2^/s, and Iohexol *D*_io_ = 250 μm^2^/s^28^. Parameters for PETE membrane, organoid and agarose were: *ε*_PETE_ = 0.094, *ε*_org_ = 0.2^29,30^, *ε*_ag_ = 0.99 for porosities; *k*_PETE_ = 1.2E-16 m^2^ ^31^, *k*_org_ = 3.75E-15 m^2^ ^29,30^, *k*_ag_ = 8.2E-16 m^2^ ^32^ for permeabilities.

### MicroCT

MicroCT imaging work was performed at the Northwestern University Center for Advanced Molecular Imaging (RRID:SCR_021192) generously supported by NCI CCSG P30 CA060553 awarded to the Robert H Lurie Comprehensive Cancer Center. The diffusion characteristics of the device were evaluated using a preclinical microCT system (Bruker Skyscan 1276, Bruker Belgium, Kontich, Belgium). The scanning parameters were set with source current 200 mA, source voltage 55 kV using a 0.25 mm Al filter, 2 × 2 binning, 1,000 projections over 360°, 2 frame averages, and an isotropic voxel size of 18.56 mm. The chamber well was filled with 1% agarose to mimic the physical environment of the device when embedded in an organoid. The microfluidic channels were perfused with an aqueous iohexol solution at a concentration of 300 mg/mL, with flow driven by hydrostatic pressure. After 24 h, a higher resolution scan was performed with 1 × 1 binning, 1,028 projections, and an isotropic voxel size of 9.3 mm. Images were reconstructed with filtered backprojection using a Hamming filter (Alpha=0.54), NRecon v2.2.0.6 (Bruker Belgium, Kontich, Belgium).

### hESC culture

HUES 3 Hb9::GFP (RRID:CVCL_X724) was obtained from the HSCI (Harvard Stem Cell Institute) iPS Core Facility, Harvard University. This transgenic hESC line stably expresses GFP under the control of the murine Hb9 promoter, enabling real-time identification of motoneurons during differentiation^33^. hESCs were expanded under feeder-free conditions using mTsER Plus (100-0276, StemCell Technologies) in Vitronectin (0.5 μg/ml; A14700, Thermo Fisher Scientific) coated 6-well plates. Cells were passaged using ReLeSR (100-0484, Stem Cell Technologies) every 4-5 days. Cells from passage numbers 21-44 were used for all experiments.

### Generation of spinal cord organoids

Human spinal cord organoids were generated by adapting established protocols^33^. Briefly, upon 80% confluency of hESCs, cells were dissociated using accutase (50-112-9055, Thermo Fisher Scientific), followed by resuspension and centrifugation (200g, 4 min) in mTsER Plus supplemented with ROCK inhibitor (10μM; 129830-38-2, DNSK International). Cells were then plated at a density of 10,000 cells per microwell in an AggreWell 800 plate (34815, StemCell Technologies) previously coated with anti-adherence rinsing solution (07010, Stem Cell Technologies). The plate was then centrifuged at 100 g for 3 min to facilitate self-formation of spherical structures. After 24 h, organoids were dislodged and maintained in ultra-low attachment plates prior to transfer to devices on day 7 of differentiation. From day 0-5, base medium consisted of mTsER Plus supplemented with SMAD inhibitors dorsomorphin (5 μM; 171261, EMD Millipore) and SB431542 (10 μM; 301836-41-9, DNSK International). Starting on day 4, medium was supplemented with WNT signaling pathway activator CHIR 99021 until day 17(3 μM; 4423, Tocris). From day 6-19 base differentiation medium changed to 96% Neurobasal-A (10-888-022, Thermo Fisher Scientific), 2% B-27 (12-587-001, Thermo Fisher Scientific), 1% GlutaMAX (35-050-061, Thermo Fisher Scientific), 1% Penicillin-Streptomycin (10,000 U/ml; 15-140-122, Thermo Fisher Scientific). Retinoic acid (0.1 μM; R2625-50MG, Sigma Aldrich) and epidermal growth factor (EGF, 20 ng/ml; 236-EG-200, R&D) were added from day 6 to 17 for neural induction. Organoids were transferred to microfluidic or control devices between day 7-9 of differentiation. Smoothened agonist, a sonic hedgehog modulator, (SAG, 1 μM; 566660, Millipore) was added from day 11 to 17. From day 19 onwards, base medium was additionally supplemented with 1% N2 (17-502-001, Thermo Fisher Scientific), brain derived neurotrophic factor (BDNF, 20 ng/ml; 248-BDB-250/CF, R&D Systems), insulin-like growth factor 1 (IGF-1, 10 ng/ml; 100-11, Peprotech), ascorbic acid (AA, 200n; MA4403, Sigma Aldrich), and cyclic adenosine monophosphate (cAMP, 200 nM; D0627, Sigma-Aldrich). Lastly, N-[N-(3,5-difluorophenacetyl)-l-alanyl]-S-phenylglycine t-butyl ester (DAPT, 2.5 μM; 2634-10, Tocris) was introduced on day 19-23 for inhibition of Notch signaling. Media changes occurred daily for the first 6 days, followed by every other day until the day prior to fixation, and cultures were kept in a 37 °C incubator with 5% CO_2._ See Extended Data Fig. 3.a for visual timeline of organoid formation protocol.

### Device Sterilization

Prior to organoid-microfluidic device integration, all devices underwent plasma treatment using high power expanded plasma cleaner (PDC-001-HP, Harrick Plasma) at 45 W for 5 min controlled by a dry scroll pump (IDP3, Agilent). Following surface treatment and removal of residual nanoscale organic contamination, devices underwent 20-min UV treatment under a class II-A2 biological safety cabinet. With unfiltered 200 μl pipette tip connected to device inlet ports, 70% ethanol was delivered using syringe and 25G needle until flow was visible in the outlet port. Then, a 1 ml Air-Tite sterile syringe with Luer lock tip (14-817-108, Thermo Fisher Scientific) was connected to inlet ports and 0.1 ml were flushed holding syringe at 0.9 ml volume for 1 min to ensure proper flow in microchannels. To avoid deformation of device wing structures, the chamber was filled with DI H_2_O immediately after 70% ethanol flush. Samples remained under this condition for 15 min, ensuring no evaporation to avoid devices drying out. Upon incubation, both device chamber and microchannels were flushed with DI H_2_O to wash off any remaining 70% ethanol. For the chamber, three half volume changes were performed with DI H_2_O. For microchannels, a new 200 μl unfiltered pipette tip was connected to the inlet port and 3 ml of DI H_2_O were flushed as in previous steps. Finally, prior to organoid insertion, a final flush with 37 °C Dulbecco’s Modified Eagle Medium (DMEM, 1885084, Gibco) was performed in microchannels to visualize any air bubbles under sterile conditions using a digital microscope (VHX-X1, Keyence). All microfluidic control samples were sterilized following only chamber steps.

### Integration of organoid with 3D fluidic devices and culture maintenance

Upon sterilization, DMEM was replaced with base medium supplemented with morphogens (day 7). Devices were then opened by gently pressing the elastomer lower layer upwards to facilitate cage structure opening for organoid insertion. Day-7 organoids were transferred using wide bore filtered p-200 μl pipette tips (2069G, Thermo Scientific ART). Organoids were maintained in media as mentioned in the organoid generation methods section. During media changes, three half volume media changes were performed to avoid disruption of organoid-device integration. Volume flowing through the microchannels was changed by performing a 1 ml flush through the inlet port via syringe, followed by 0.1 ml flush for 30 s using 1 ml Air-Tite sterile syringe. A 1ml unfiltered tip (6.4 cm height; F171500, Gilson) was connected to the inlet port and the volume was filled to 1.2 ml with supplemented media to allow for sufficient hydrostatic pressure to establish efficient flow through the microfluidic channel. Live imaging was performed on every media-change day using an automated upright microscope (DM6000B, Leica) and digital microscope (VHX-X1, Keyence) in a biological safety cabinet for growth tracking measuring organoid diameter and circumference for size characterization.

### MRI and CT imaging

MRI and CT were performed in the CTI Small Animal Molecular Imaging core facility (RRID:SCR_017878) at Northwestern University. Computed tomography imaging was performed on a NanoScan 8 (Mediso, Hungary) with X-ray power set at 50Kvp and 600 mA, 720 projections and 0.04 x 0.04 x 0.04 mm resolution. MR images were acquired on a 7T Biospec Avance Neo (Bruker, Germany). 3D T1 weighted images were acquired with TE:8.6 ms, TR:40 ms, FA=20, NEX:6, slice thickness: 4.4 mm, matrix:192x192x12. T1 maps were obtained by multiple angle 2D FLASH with TE:3.5 ms, TR:204 ms, NEX:3, slices:5, slice thickness: 0.6 mm, matrix: 256x256. Eight scans were performed with FA ranging from angles of 10 to 90 degrees. Acquisitions were repeated following 1 h incubation in 5 mM Magnevist (Bayer, Germany) and 3x washes with 1X PBS through microfluidic wings and chamber. DICOM files were used for image analysis using the different software packages including VivoQuant 4.0 (Perceptive, MA), ITK-SNAP4, Jim 9.0 (Xinapse Systems Ltd, UK) and MeshLab (Visual Computing Lab of ISTI-CNR)5. CT 3D renderings were obtained in VivoQuant. MRI 3D renderings were obtained using semi-automated thresholding based on T1weighted contrast to identify 3 regions of high (top 33%), medium and low (lower 33%) contrast to identify contrast agent diffusion. The resulting segmented regions were transformed into STL format and visualized using MeshLab2022. T1 maps were obtained in Jim 9.0.

### Morphogen delivery assay validation

To validate delivery capability of microfluidic platforms, a group of organoid-microfluidic platforms were fed only with base medium (Neurobasal-A, N2, B27, P/S and GlutaMAX) and no morphogens throughout differentiation protocol in both chamber and microchannels, serving as a negative control group. A second group was fed with morphogens only in the microchannels and no morphogens in the chamber, serving as a positive control group (Fig. 3c). A third group of microfluidic samples was fed following regular procedures.

### Selective neuronal labeling with CTB delivery assay

CTB-555 conjugate (500 μg/ml; C22843, Invitrogen) was used to selectively label neurons in microenvironments surrounding microchannels in spinal cord organoids at 1:100. Fluidic devices were flushed with 250 μl of CTB using a p-200 unfiltered pipetted tip with syringe, followed by a 30 s flush via 1 ml syringe. Volume was kept at a 500 μg/ml concentration for 2 h. Afterwards, p-1000 μl unfiltered pipette tips were connected and filled with 1260 μl of cell maintenance media to achieve the hydrostatic flow needed to facilitate flow through the channels for 48 h. Organoids were then fixed using 4% paraformaldehyde for 1 h at room temperature.

### Post-fixation dye delivery

For nuclear dye multiplexed delivery, organoids were fixed using 4% paraformaldehyde for 1 h at room temperature. Incubation of 4′,6-diamidino-2-phenylindole (DAPI) at 1:50 (1μg/ml; D1306, Invitrogen) and DRAQ5 at 1:100 (5μM; ab108410, Abcam) was performed using p-200 μl unfiltered pipetted tip connected to the inlet. A 30 s flush with a 1ml syringe was performed following dye delivery to prevent bubble formation. P-1000 μl unfiltered pipette tips were connected and filled with volume of 1260 μl to achieve the hydrostatic pressure needed to facilitate experimental flow through fluidic microchannels for 48 h at 4 °C. Sectioned samples were then mounted with antifade mounting medium (H-1700, Vector Laboratories).

### Histology

Prior to cryopreservation, organoids were placed in 30% sucrose for dehydration. Samples were then detached from the elastomer layer by cutting microfluidic wings and detaching the organoid-microfluidic from bonding sites gently. Immediately after, samples were incubated with gelatin (G2500, Sigma) dissolved in 10% sucrose solution at 37 °C for 1 h and then placed in cryomold for snap-freezing by submersion in cooled 100% ethanol. Samples were then sectioned on a cryostat (CM3050S, Leica) at 20 μm sections using step technique to accommodate for sections throughout the whole sample. For paraffin embedding, samples were fixed in 10% formalin solution, followed by triple 1X PBS washes. Samples were then dehydrated in 70% ethanol and embedded in paraffin blocks for sectioning using microtome (HM355S, Thermo Fisher Scientific). Paraffin-embedded samples were sectioned with assistance from the Northwestern University Mouse Histology and Phenotyping Laboratory.

### Organoid imaging and analysis

Sections were washed with PBS, followed by blocking for 1 h at room temperature using 0.3 vol% Triton-100X (T9010, Aqua Solution Inc.) in 1wt% bovine serum albumin (A9418, Sigma Aldrich) solution in humidified chambers. Sections were then stained with primary antibodies in blocking solution overnight in humidified chambers at 4 °C. Samples were triple washed with 1X PBS, followed by a 1 h secondary incubation of secondary antibodies in blocking solution protected from light at room temperature. A complete list of antibodies used is listed in Supplementary Table 1. All images of fixed organoids were acquired using 20 X objective, slide scanner (VS200, Olympus), digital camera (C1440, Hamamatsu Orca), light source (XT720 S, X-Cite XYLIS LED Illumination System), and the following filter cubes LED-DAPI, LED-FITC, LED-mCherry and LED Cy5 of immunofluorescence imaging, and brightfield for TUNEL staining.

Imaging analysis was performed using image analysis software FIJI (ImageJ). Labkit plugin, which uses a base pixel classification based on the Waikato Environment for Knowledge Analysis (WEKA), was used for classification analysis prior to segmentation^34^. Briefly, single channel images were used to classify pixels in the following categories: background, organoid background, device channel, or fluorescent dye of interest. Classifier algorithms were then trained until a consistent classification across multiple samples was obtained. Probability maps were then exported, and appropriate thresholds were applied to quantify the area and particle number positive for the fluorescent dye in a whole organoid image as well as selected regions of interest (250x250 μm ROIs) around device channels. For organoid outgrowth analysis, images acquired using a digital microscope (VHX-X1, Keyence) were manually measured to quantify diameter and perimeters using VHX-970F/970FN software (Keyence).

### Statistics

All statistical analysis was performed using GraphPad Prism 10.4.1. All data was checked for normality, and logarithmic transformation was performed accordingly for non-normally distributed data. For comparisons between two groups with normal distributions, 2-tailed, unpaired t-test analysis was used and values reported are means ± standard deviations. For non-normally distributed data, Mann-Whitney U test analysis was performed, and values reported are median with their corresponding interquartile ranges (Q1-Q3). P values less than 0.05 were considered statistically significant.

## Extended Data

## Supplementary Material

This is a list of supplementary files associated with this preprint. Click to download.

• QuezadaSupplementary.pdf

## Data Availability

All data is available in Source Data file.

## Code Availability

No unique code was generated in this paper.
